# Crystal structure of 4-chloro-2-[(5-eth­oxy-1,3,4-thia­diazol-2-yl)meth­yl]-5-(piperidin-1-yl)pyridazin-3(2*H*)-one

**DOI:** 10.1107/S1600536814020662

**Published:** 2014-09-20

**Authors:** Hongsen Li, Xinfeng Ren, Ya Li, Linjing Zhao

**Affiliations:** a College of Chemistry and Chemical Engineering, Shanghai University of Engineering Science, 333 Longteng Road, Shanghai, People’s Republic of China

**Keywords:** pyridazinone derivatives, crystal structure, C—H—O hydrogen bonding

## Abstract

In the title mol­ecule, C_14_H_18_ClN_5_O_2_S, the six atoms of the 1,6-di­hydro­pyridazine ring are essentially coplanar (r.m.s. deviation = 0.008 Å), and the dihedral angle between this and the 1,3,4-thia­diazole ring is 62.06 (10)°. In the crystal, centrosymmetrically related mol­ecules are linked by inter­molecular C—H—O hydrogen bonding to form a supra­molecular dimer. The terminal ethyl group is statistically disordered over two positions.

## Related literature   

For the biological activity of pyridazinone derivatives, see: Abouzid *et al.* (2008 ▶); Siddiqui *et al.* (2010 ▶), and for their synthesis, see: Wang *et al.* (2010 ▶); Zhang *et al.* (2002 ▶).
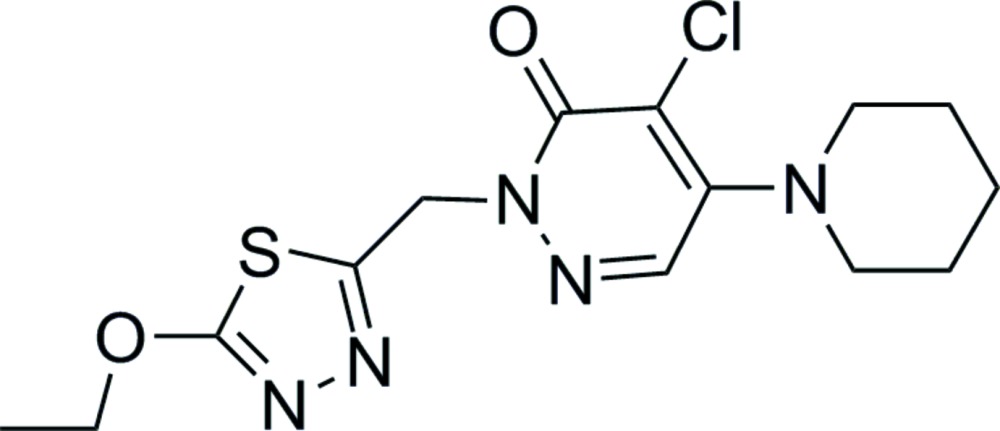


## Experimental   

### Crystal data  

C_14_H_18_ClN_5_O_2_S
*M* *_r_* = 355.84
Triclinic, 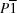

*a* = 5.2840 (8) Å
*b* = 11.0323 (16) Å
*c* = 14.902 (2) Å
α = 107.318 (2)°
β = 91.590 (2)°
γ = 99.528 (2)°
*V* = 815.1 (2) Å^3^
*Z* = 2
Mo *K*α radiation
μ = 0.38 mm^−1^
*T* = 296 K
0.30 × 0.24 × 0.16 mm

### Data collection  

Bruker APEXII CCD diffractometer
Absorption correction: multi-scan (*SADABS*; Sheldrick, 1996 ▶) *T*_min_ = 0.895, *T*_max_ = 0.942
4244 measured reflections
2828 independent reflections
2490 reflections with *I* > 2σ(*I*)
*R*_int_ = 0.012

### Refinement  

*R*[*F* ^2^ > 2σ(*F* ^2^)] = 0.039
*wR*(*F* ^2^) = 0.121
*S* = 1.58
2828 reflections
229 parameters
H-atom parameters constrained
Δρ_max_ = 0.22 e Å^−3^
Δρ_min_ = −0.23 e Å^−3^

### 

Data collection: *APEX2* (Bruker, 2009 ▶); cell refinement: *SAINT* (Bruker, 2009 ▶); data reduction: *SAINT*; program(s) used to solve structure: *SHELXS97* (Sheldrick, 2008 ▶); program(s) used to refine structure: *SHELXL97* (Sheldrick, 2008 ▶); molecular graphics: *SHELXTL* (Sheldrick, 2008 ▶); software used to prepare material for publication: *SHELXTL*.

## Supplementary Material

Crystal structure: contains datablock(s) I. DOI: 10.1107/S1600536814020662/tk5340sup1.cif

Structure factors: contains datablock(s) I. DOI: 10.1107/S1600536814020662/tk5340Isup2.hkl

Supporting information file. DOI: 10.1107/S1600536814020662/tk5340Isup3.cml

**Fig. 1.**
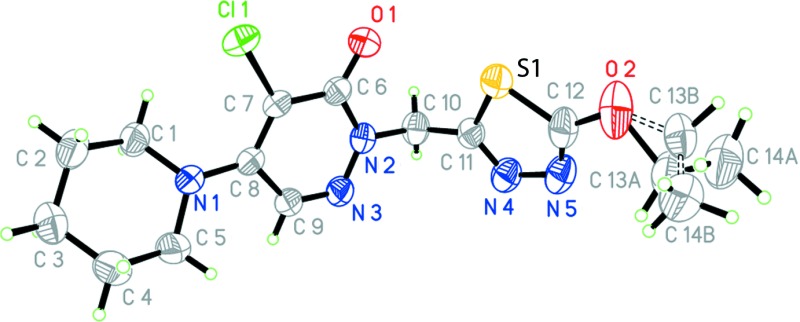
Molecular structure of the title compound showing atom labelling and displacement ellipsoids at 50%.

. DOI: 10.1107/S1600536814020662/tk5340fig1.tif
Mol­ecular structure of the title compound showing atom labelling and displacement ellipsoids at 50%.

CCDC reference: 1024313

Additional supporting information:  crystallographic information; 3D view; checkCIF report

## Figures and Tables

**Table 1:** Hydrogen-bond geometry (Å, °)

*D*—H⋯*A*	*D*—H	H⋯*A*	*D*⋯*A*	*D*—H⋯*A*
C14*A*—H14*A*⋯O1^i^	0.96	2.45	3.366 (11)	160

Symmetry code: (i) 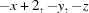.

## supplementary crystallographic information

### S2. Experimental

A mixture of 4,5-di­chloro-2-[(5-eth­oxy-1,3,4-thia­diazol-2-yl)methyl]-pyridazin-3(2H)-one (3.98 g, 1.3 mmol), piperidine (1.37 g, 19.5 mmol), potassium carbonate (3 g) and dry DMF (30mL) was stirred at 40^o^C for 8 h. The mixture was then poured into ice-water and a yellow precipitate -formed. The precipitate was washed with water, followed by vaccum drying, to give the pure title compound (3.38 g, yield: 73.2 %). The obtained compound was recrystallized from its ethyl acetate/petroleum ether (5:1) to give yellow crystals.

### S2.1. Refinement

Carbon-bound H-atoms were placed in calculated positions (C—H = 0.93 to 0.97 Å) and were included in the refinement in the riding model approximation, with *U_iso_*(H) = 1.2–1.5*U_eq_*(C). The terminal ethyl group (C13 and C14) was statistically disordered over two positions.

### Crystal data

**Table:**

C_14_H_18_ClN_5_O_2_S	*Z* = 2
*M**_r_* = 355.84	*F*(000) = 372
Triclinic, *P*1	*D*_x_ = 1.450 Mg m^−^^3^
Hall symbol: -P 1	Mo *K*α radiation, λ = 0.71073 Å
*a* = 5.2840 (8) Å	Cell parameters from 2456 reflections
*b* = 11.0323 (16) Å	θ = 2.8–27.3°
*c* = 14.902 (2) Å	µ = 0.38 mm^−^^1^
α = 107.318 (2)°	*T* = 296 K
β = 91.590 (2)°	Block, yellow
γ = 99.528 (2)°	0.30 × 0.24 × 0.16 mm
*V* = 815.1 (2) Å^3^	

### Data collection

**Table:**

Bruker APEXII CCD diffractometer	2828 independent reflections
Radiation source: fine-focus sealed tube	2490 reflections with *I* > 2σ(*I*)
Graphite monochromator	*R*_int_ = 0.012
φ and ω scans	θ_max_ = 25.1°, θ_min_ = 2.0°
Absorption correction: multi-scan (*SADABS*; Sheldrick, 1996)	*h* = −6→6
*T*_min_ = 0.895, *T*_max_ = 0.942	*k* = −13→13
4244 measured reflections	*l* = −13→17

### Refinement

**Table:**

Refinement on *F*^2^	Primary atom site location: structure-invariant direct methods
Least-squares matrix: full	Secondary atom site location: difference Fourier map
*R*[*F*^2^ > 2σ(*F*^2^)] = 0.039	Hydrogen site location: inferred from neighbouring sites
*wR*(*F*^2^) = 0.121	H-atom parameters constrained
*S* = 1.58	*w* = 1/[σ^2^(*F*_o_^2^) + (0.0546*P*)^2^] where *P* = (*F*_o_^2^ + 2*F*_c_^2^)/3
2828 reflections	(Δ/σ)_max_ = 0.050
229 parameters	Δρ_max_ = 0.22 e Å^−^^3^
0 restraints	Δρ_min_ = −0.23 e Å^−^^3^

### Special details

**Table:**

Geometry. All e.s.d.'s (except the e.s.d. in the dihedral angle between two l.s. planes) are estimated using the full covariance matrix. The cell e.s.d.'s are taken into account individually in the estimation of e.s.d.'s in distances, angles and torsion angles; correlations between e.s.d.'s in cell parameters are only used when they are defined by crystal symmetry. An approximate (isotropic) treatment of cell e.s.d.'s is used for estimating e.s.d.'s involving l.s. planes.
Refinement. Refinement of *F*^2^ against ALL reflections. The weighted *R*-factor *wR* and goodness of fit *S* are based on *F*^2^, conventional *R*-factors *R* are based on *F*, with *F* set to zero for negative *F*^2^. The threshold expression of *F*^2^ > σ(*F*^2^) is used only for calculating *R*-factors(gt) *etc*. and is not relevant to the choice of reflections for refinement. *R*-factors based on *F*^2^ are statistically about twice as large as those based on *F*, and *R*- factors based on ALL data will be even larger.

### Fractional atomic coordinates and isotropic or equivalent isotropic displacement parameters (Å^2^)

**Table:**

	*x*	*y*	*z*	*U*_iso_*/*U*_eq_	Occ. (<1)
C1	1.1293 (4)	0.40442 (18)	0.64144 (14)	0.0529 (5)	
H1A	0.9963	0.4362	0.6807	0.064*	
H1B	1.1620	0.4550	0.5984	0.064*	
C2	1.3686 (5)	0.4198 (2)	0.70167 (19)	0.0710 (7)	
H2A	1.4236	0.5100	0.7376	0.085*	
H2B	1.5043	0.3928	0.6621	0.085*	
C3	1.3239 (5)	0.3396 (2)	0.76876 (18)	0.0726 (7)	
H3A	1.4850	0.3448	0.8038	0.087*	
H3B	1.2037	0.3733	0.8135	0.087*	
C4	1.2172 (5)	0.2004 (2)	0.71378 (17)	0.0623 (6)	
H4A	1.3494	0.1633	0.6768	0.075*	
H4B	1.1719	0.1521	0.7576	0.075*	
C5	0.9873 (5)	0.1870 (2)	0.65010 (17)	0.0654 (6)	
H5A	0.9358	0.0973	0.6122	0.079*	
H5B	0.8461	0.2120	0.6873	0.079*	
C6	0.8041 (4)	0.24108 (16)	0.34196 (13)	0.0418 (4)	
C7	0.9639 (3)	0.28093 (16)	0.42889 (13)	0.0390 (4)	
C8	0.8999 (4)	0.23463 (16)	0.50280 (13)	0.0426 (4)	
C9	0.6649 (4)	0.14118 (19)	0.48425 (14)	0.0513 (5)	
H9	0.6167	0.1065	0.5323	0.062*	
C10	0.3987 (4)	0.1095 (2)	0.25530 (14)	0.0517 (5)	
H10A	0.3853	0.1833	0.2340	0.062*	
H10B	0.2307	0.0783	0.2729	0.062*	
C11	0.4730 (4)	0.00539 (18)	0.17587 (13)	0.0452 (5)	
C12	0.6399 (5)	−0.1324 (2)	0.05014 (15)	0.0605 (6)	
C13A	0.6909 (17)	−0.3381 (7)	−0.0461 (7)	0.074 (2)	0.503 (13)
H13A	0.6744	−0.3654	0.0099	0.089*	0.503 (13)
H13B	0.5282	−0.3668	−0.0845	0.089*	0.503 (13)
C14A	0.9045 (12)	−0.3878 (6)	−0.1004 (7)	0.083 (3)	0.503 (13)
H14A	0.9387	−0.3461	−0.1480	0.125*	0.503 (13)
H14B	0.8561	−0.4794	−0.1298	0.125*	0.503 (13)
H14C	1.0565	−0.3701	−0.0584	0.125*	0.503 (13)
C13B	0.7259 (16)	−0.3012 (8)	−0.0838 (6)	0.071 (2)	0.497 (13)
H13C	0.8075	−0.3014	−0.1413	0.085*	0.497 (13)
H13D	0.5414	−0.3283	−0.0987	0.085*	0.497 (13)
C14B	0.8311 (17)	−0.3870 (7)	−0.0377 (8)	0.086 (3)	0.497 (13)
H14D	1.0040	−0.3483	−0.0114	0.129*	0.497 (13)
H14E	0.8312	−0.4692	−0.0836	0.129*	0.497 (13)
H14F	0.7260	−0.3987	0.0116	0.129*	0.497 (13)
Cl1	1.24862 (9)	0.38323 (4)	0.42952 (4)	0.0529 (2)	
N1	1.0406 (3)	0.26824 (15)	0.58761 (11)	0.0507 (4)	
N2	0.5837 (3)	0.15084 (14)	0.33835 (11)	0.0437 (4)	
N3	0.5152 (3)	0.10080 (16)	0.40812 (12)	0.0519 (4)	
N4	0.3405 (4)	−0.10968 (17)	0.14941 (13)	0.0585 (5)	
N5	0.4391 (4)	−0.19254 (17)	0.07519 (14)	0.0630 (5)	
O1	0.8484 (3)	0.27791 (13)	0.27325 (10)	0.0585 (4)	
O2	0.7819 (4)	−0.1833 (2)	−0.01905 (13)	0.0901 (6)	
S1	0.73552 (11)	0.02873 (5)	0.11291 (4)	0.0556 (2)	

### Atomic displacement parameters (Å^2^)

**Table:**

	*U*^11^	*U*^22^	*U*^33^	*U*^12^	*U*^13^	*U*^23^
C1	0.0616 (13)	0.0406 (11)	0.0504 (12)	0.0058 (9)	−0.0067 (10)	0.0075 (9)
C2	0.0735 (16)	0.0530 (13)	0.0755 (16)	−0.0080 (11)	−0.0252 (13)	0.0164 (12)
C3	0.0772 (17)	0.0714 (15)	0.0639 (15)	0.0039 (12)	−0.0229 (13)	0.0205 (13)
C4	0.0673 (15)	0.0634 (14)	0.0622 (14)	0.0080 (11)	−0.0004 (11)	0.0307 (12)
C5	0.0680 (15)	0.0626 (14)	0.0651 (14)	−0.0112 (11)	−0.0107 (12)	0.0326 (12)
C6	0.0471 (11)	0.0336 (9)	0.0436 (11)	0.0097 (8)	0.0044 (8)	0.0091 (8)
C7	0.0404 (10)	0.0292 (8)	0.0459 (10)	0.0035 (7)	0.0023 (8)	0.0109 (7)
C8	0.0475 (11)	0.0324 (9)	0.0443 (11)	0.0013 (7)	−0.0012 (8)	0.0102 (8)
C9	0.0562 (13)	0.0474 (11)	0.0436 (11)	−0.0099 (9)	−0.0005 (9)	0.0145 (9)
C10	0.0438 (11)	0.0568 (12)	0.0496 (12)	0.0116 (9)	−0.0059 (9)	0.0085 (9)
C11	0.0427 (11)	0.0483 (11)	0.0413 (10)	0.0047 (8)	−0.0079 (8)	0.0117 (8)
C12	0.0638 (15)	0.0613 (13)	0.0476 (12)	0.0168 (11)	−0.0100 (11)	0.0022 (10)
C13A	0.088 (5)	0.058 (4)	0.058 (5)	0.008 (3)	0.015 (4)	−0.006 (3)
C14A	0.073 (4)	0.068 (4)	0.088 (6)	0.010 (3)	0.024 (4)	−0.007 (3)
C13B	0.080 (4)	0.073 (5)	0.045 (4)	0.009 (3)	−0.002 (3)	0.000 (3)
C14B	0.090 (6)	0.075 (4)	0.085 (6)	0.010 (4)	−0.001 (5)	0.017 (4)
Cl1	0.0456 (3)	0.0449 (3)	0.0658 (4)	−0.0037 (2)	0.0049 (2)	0.0198 (2)
N1	0.0600 (11)	0.0404 (9)	0.0476 (10)	−0.0061 (7)	−0.0102 (8)	0.0170 (7)
N2	0.0423 (9)	0.0424 (8)	0.0409 (9)	0.0042 (7)	−0.0018 (7)	0.0069 (7)
N3	0.0518 (10)	0.0487 (9)	0.0467 (10)	−0.0064 (7)	0.0007 (8)	0.0107 (8)
N4	0.0539 (11)	0.0553 (11)	0.0567 (11)	−0.0004 (8)	−0.0072 (9)	0.0092 (9)
N5	0.0615 (12)	0.0521 (11)	0.0610 (12)	0.0042 (9)	−0.0115 (10)	0.0007 (9)
O1	0.0772 (10)	0.0514 (8)	0.0479 (8)	0.0030 (7)	−0.0008 (7)	0.0220 (7)
O2	0.0843 (13)	0.0960 (14)	0.0663 (11)	0.0270 (11)	0.0062 (10)	−0.0163 (10)
S1	0.0575 (4)	0.0545 (3)	0.0502 (3)	0.0046 (2)	0.0026 (3)	0.0120 (2)

### Geometric parameters (Å, º)

**Table:**

C1—N1	1.466 (2)	C10—N2	1.464 (2)
C1—C2	1.486 (3)	C10—C11	1.496 (3)
C1—H1A	0.9700	C10—H10A	0.9700
C1—H1B	0.9700	C10—H10B	0.9700
C2—C3	1.520 (3)	C11—N4	1.284 (3)
C2—H2A	0.9700	C11—S1	1.723 (2)
C2—H2B	0.9700	C12—N5	1.282 (3)
C3—C4	1.512 (3)	C12—O2	1.334 (3)
C3—H3A	0.9700	C12—S1	1.725 (2)
C3—H3B	0.9700	C13A—C14A	1.491 (13)
C4—C5	1.480 (3)	C13A—O2	1.619 (8)
C4—H4A	0.9700	C13A—H13A	0.9700
C4—H4B	0.9700	C13A—H13B	0.9700
C5—N1	1.476 (2)	C14A—H14A	0.9600
C5—H5A	0.9700	C14A—H14B	0.9600
C5—H5B	0.9700	C14A—H14C	0.9600
C6—O1	1.224 (2)	C13B—O2	1.349 (7)
C6—N2	1.390 (2)	C13B—C14B	1.489 (14)
C6—C7	1.436 (3)	C13B—H13C	0.9700
C7—C8	1.374 (3)	C13B—H13D	0.9700
C7—Cl1	1.7228 (18)	C14B—H14D	0.9600
C8—N1	1.366 (2)	C14B—H14E	0.9600
C8—C9	1.438 (3)	C14B—H14F	0.9600
C9—N3	1.282 (3)	N2—N3	1.347 (2)
C9—H9	0.9300	N4—N5	1.385 (3)
			
N1—C1—C2	110.36 (17)	N2—C10—H10A	109.0
N1—C1—H1A	109.6	C11—C10—H10A	109.0
C2—C1—H1A	109.6	N2—C10—H10B	109.0
N1—C1—H1B	109.6	C11—C10—H10B	109.0
C2—C1—H1B	109.6	H10A—C10—H10B	107.8
H1A—C1—H1B	108.1	N4—C11—C10	121.30 (19)
C1—C2—C3	110.7 (2)	N4—C11—S1	114.96 (16)
C1—C2—H2A	109.5	C10—C11—S1	123.74 (14)
C3—C2—H2A	109.5	N5—C12—O2	126.0 (2)
C1—C2—H2B	109.5	N5—C12—S1	116.59 (17)
C3—C2—H2B	109.5	O2—C12—S1	117.4 (2)
H2A—C2—H2B	108.1	C14A—C13A—O2	102.4 (7)
C4—C3—C2	109.89 (19)	C14A—C13A—H13A	111.3
C4—C3—H3A	109.7	O2—C13A—H13A	111.3
C2—C3—H3A	109.7	C14A—C13A—H13B	111.3
C4—C3—H3B	109.7	O2—C13A—H13B	111.3
C2—C3—H3B	109.7	H13A—C13A—H13B	109.2
H3A—C3—H3B	108.2	O2—C13B—C14B	104.2 (7)
C5—C4—C3	112.46 (19)	O2—C13B—H13C	110.9
C5—C4—H4A	109.1	C14B—C13B—H13C	110.9
C3—C4—H4A	109.1	O2—C13B—H13D	110.9
C5—C4—H4B	109.1	C14B—C13B—H13D	110.9
C3—C4—H4B	109.1	H13C—C13B—H13D	108.9
H4A—C4—H4B	107.8	C13B—C14B—H14D	109.5
N1—C5—C4	111.07 (17)	C13B—C14B—H14E	109.5
N1—C5—H5A	109.4	H14D—C14B—H14E	109.5
C4—C5—H5A	109.4	C13B—C14B—H14F	109.5
N1—C5—H5B	109.4	H14D—C14B—H14F	109.5
C4—C5—H5B	109.4	H14E—C14B—H14F	109.5
H5A—C5—H5B	108.0	C8—N1—C1	120.60 (15)
O1—C6—N2	119.39 (17)	C8—N1—C5	119.35 (15)
O1—C6—C7	125.93 (18)	C1—N1—C5	111.72 (16)
N2—C6—C7	114.67 (16)	N3—N2—C6	125.32 (15)
C8—C7—C6	122.23 (17)	N3—N2—C10	115.33 (15)
C8—C7—Cl1	123.29 (14)	C6—N2—C10	119.26 (16)
C6—C7—Cl1	114.37 (14)	C9—N3—N2	116.97 (16)
N1—C8—C7	125.69 (17)	C11—N4—N5	113.00 (19)
N1—C8—C9	120.06 (17)	C12—N5—N4	110.34 (17)
C7—C8—C9	114.23 (17)	C12—O2—C13B	127.2 (5)
N3—C9—C8	126.57 (19)	C12—O2—C13A	105.7 (4)
N3—C9—H9	116.7	C13B—O2—C13A	29.3 (3)
C8—C9—H9	116.7	C11—S1—C12	85.12 (11)
N2—C10—C11	112.73 (15)		
			
N1—C1—C2—C3	−58.3 (3)	C7—C6—N2—N3	−0.2 (3)
C1—C2—C3—C4	54.5 (3)	O1—C6—N2—C10	−4.9 (3)
C2—C3—C4—C5	−52.6 (3)	C7—C6—N2—C10	176.21 (15)
C3—C4—C5—N1	53.7 (3)	C11—C10—N2—N3	−100.33 (19)
O1—C6—C7—C8	−179.95 (18)	C11—C10—N2—C6	82.9 (2)
N2—C6—C7—C8	−1.1 (3)	C8—C9—N3—N2	−0.1 (3)
O1—C6—C7—Cl1	−3.6 (2)	C6—N2—N3—C9	0.8 (3)
N2—C6—C7—Cl1	175.26 (12)	C10—N2—N3—C9	−175.75 (18)
C6—C7—C8—N1	−179.63 (17)	C10—C11—N4—N5	−179.86 (16)
Cl1—C7—C8—N1	4.3 (3)	S1—C11—N4—N5	0.5 (2)
C6—C7—C8—C9	1.6 (3)	O2—C12—N5—N4	180.0 (2)
Cl1—C7—C8—C9	−174.41 (14)	S1—C12—N5—N4	0.7 (2)
N1—C8—C9—N3	−179.9 (2)	C11—N4—N5—C12	−0.8 (3)
C7—C8—C9—N3	−1.0 (3)	N5—C12—O2—C13B	9.5 (7)
N2—C10—C11—N4	112.2 (2)	S1—C12—O2—C13B	−171.2 (5)
N2—C10—C11—S1	−68.2 (2)	N5—C12—O2—C13A	−13.1 (5)
C7—C8—N1—C1	50.6 (3)	S1—C12—O2—C13A	166.2 (4)
C9—C8—N1—C1	−130.8 (2)	C14B—C13B—O2—C12	−87.8 (7)
C7—C8—N1—C5	−163.8 (2)	C14B—C13B—O2—C13A	−38.9 (11)
C9—C8—N1—C5	14.9 (3)	C14A—C13A—O2—C12	−163.5 (7)
C2—C1—N1—C8	−152.6 (2)	C14A—C13A—O2—C13B	55.1 (11)
C2—C1—N1—C5	59.4 (2)	N4—C11—S1—C12	−0.12 (17)
C4—C5—N1—C8	154.6 (2)	C10—C11—S1—C12	−179.72 (17)
C4—C5—N1—C1	−56.8 (3)	N5—C12—S1—C11	−0.36 (18)
O1—C6—N2—N3	178.74 (16)	O2—C12—S1—C11	−179.71 (19)

### Hydrogen-bond geometry (Å, º)

**Table:**

*D*—H···*A*	*D*—H	H···*A*	*D*···*A*	*D*—H···*A*
C14*A*—H14*A*···O1^i^	0.96	2.45	3.366 (11)	160

Symmetry code: (i) −*x*+2, −*y*, −*z*.
