# Supplementary figures and images for: Crystal structure of 4-chloro-2-[(5-eth­oxy-1,3,4-thia­diazol-2-yl)meth­yl]-5-(piperidin-1-yl)pyridazin-3(2H)-one
Source: Acta Crystallogr Sect E Struct Rep Online. 2014 Sep 20;70(Pt 10):o1113. doi: 10.1107/S1600536814020662 (PMC4257213; doi:10.1107/S1600536814020662)

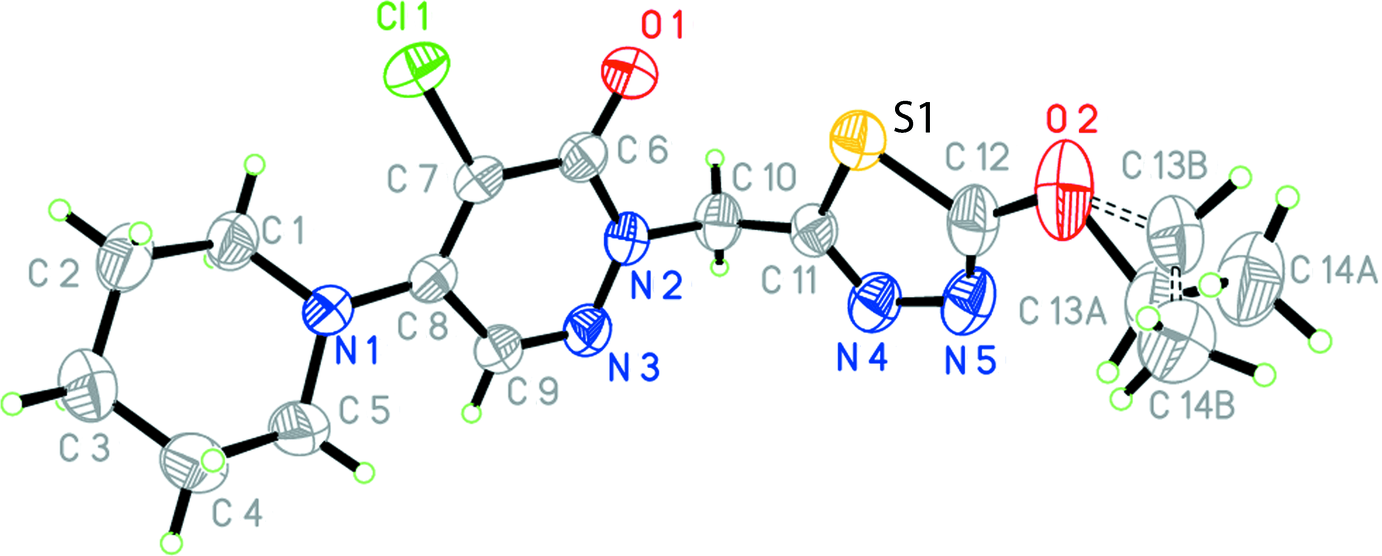

Supplement: Supplementary file 4 [file e-70-o1113-fig1.tif]
